# Identification of Circulating Exosomal microRNAs Associated with Radioiodine Refractory in Papillary Thyroid Carcinoma

**DOI:** 10.3390/jpm12122017

**Published:** 2022-12-07

**Authors:** Genpeng Li, Wenjie Chen, Yanping Gong, Tao Wei, Rixiang Gong, Jingqiang Zhu, Zhihui Li, Jianyong Lei

**Affiliations:** 1 Thyroid Surgery Center, West China Hospital of Sichuan University, Chengdu 610041, China; 2 The Laboratory of Thyroid and Parathyroid Disease, Frontiers Science Center for Disease-Related Molecular Network, West China Hospital of Sichuan University, Chengdu 610041, China; 3 Thyroid Surgery Center, West China Tianfu Hospital, Chengdu 610041, China

**Keywords:** circulating exosomal microRNAs, papillary thyroid carcinoma, radioiodine-refractory

## Abstract

Papillary thyroid carcinoma (PTC) has a favorable prognosis, but a fraction of cases show progressive behaviors, becoming radioiodine refractory (RAIR) PTC. To explore circulating exosomal microRNAs (miRNAs) associated with RAIR PTC, the miRNA profiles in exosomes from parental and induced RAIR cell lines were firstly identified with a next-generation sequencing technique. The Na^+^/I^−^ symporter (NIS) related miRNAs were then validated by quantitative real-time PCR (qRT-PCR) in plasma of PTC patients with non-^131^I-avid metastases and those with ^131^I-avid metastases. The regulation of exosomal miRNAs on NIS were also verified. We identified that miR-1296-5p, upregulation in exosomes from RAIR cell lines, and the plasma of patients with RAIR PTC achieved the largest areas under the curve (AUC) of 0.911 and that it is an independent risk factor for RAIR PTC. In addition, miR-1296-5p was abundantly detected in the tissue of RAIR PTC and can directly target downstream gene of NIS. Taken together, our findings suggested that circulating exosomal miRNAs, particularly miR-1296-5p, may be involved in the pathogenesis of RAIR PTC by directly targeting NIS.

## 1. Introduction

Papillary thyroid carcinoma (PTC), the most common subtype of thyroid cancer, has an excellent prognosis following conventional treatments, including thyroidectomy, thyroid stimulating hormone (TSH) suppression, and radioiodine (RAI) ablation [1,2]. Unfortunately, approximately 30% of PTC patients with local/distant metastasis do not take up sufficient RAI for effective ablation, which remains a problem that needs to be solved urgently [3,4,5]. It has been reported that patients with radioiodine uptake (RAIU) have a survival rate of approximately 56% at 10 years from the time of detection of metastasis, but only 10% for patients without any significant RAIU [6]. Effective treatment strategies are urgently needed to overcome the poor prognosis of these radioiodine-refractory (RAIR) PTC.

There have been several advances in understanding of the molecular pathways involved in PTC progression, such as genetic mutations (BRAF, TERT) and immunological characteristics of tumor microenvironment, which has led to the approval of tyrosine kinase inhibitors (TKIs) and immune checkpoint inhibitors in the past decades [7,8,9]. However, these therapies generally presented with low effective rates, settling on average at about 20%, and are associated with significant systemic toxicity [10,11,12,13]. Recently, accumulating evidence has suggested that exosomal microRNAs (miRNAs), a major class of small noncoding RNAs that are 17–24 nt long and abundant in exosomes, may serve as new and potentially powerful targets for therapeutic interventions against various human diseases, particularly in the field of oncology [14,15,16].

According to the 2015 American Thyroid Association (ATA) management guidelines [2], losing the ability to concentrate radioiodine in metastatic sites after previous evidence of radioiodine-avid disease is one of basic mechanisms for the development of RAIR PTC. Thus, in the present study, we mimicked the dedifferentiation of the PTC cell lines by ^131^I pretreatment according to a previous report [17] and performed next-generation sequencing (NGS) to identify differentially expressed miRNAs in exosomes. Then, we detected significant miRNAs related to the Na^+^/I^−^ symporter (NIS) by quantitative real-time PCR (qRT-PCR) in circulating exosomes between PTC patients with non-^131^I-avid metastases and those with ^131^I-avid metastases. Taken together, we found that the miR-1296-5p, one of the significantly upregulated exosomal miRNAs in circulating system in patients with RAIR PTC, can directly targets the downstream gene of NIS which may be involved in the pathogenesis of RAIR PTC.

## 2. Materials and Methods

### 2.1. Cell Lines and Patient Samples

Two human PTC cell lines, K1 and BCPAP, were purchased from Cellcook Biotech Co., Ltd. (Guangzhou, China) and Fenghui Biotech Co., Ltd. (Changsha, China). K1 cells were cultured in Dulbecco’s modified Eagle’s medium (DMEM; Invitrogen Life Technologies, CA, USA), and BCPAP cells were cultured in RPMI-1640 (Invitrogen Life Technologies, CA, USA). All media were supplemented with 10% fetal bovine serum (FBS) and 1% penicillin/streptomycin (Invitrogen Life Technologies), and the cells were grown at 5% CO_2_ and 37 °C.

Patients with the following inclusion criteria were included in the study: (1) pathologically confirmed PTC in the primary tumor, (2) total thyroidectomy, (3) any metastatic site, such as lymph node, lung, and bone, (4) history of ^131^I therapy, and (5) no history of other tumors. The enrolled patients were divided into two groups according to post-therapeutic ^131^I whole-body scans: a non-^131^I-avid group and a ^131^I-avid metastases group. Ten milliliters of venous blood from each participant were centrifuged at 1800× *g* for 10 min at 4 °C within 2 h of collection, followed by a second centrifugation at 3000× *g* for 15 min at 4 °C to eliminate any residual cell debris. The supernatant serum was then stored at −80 °C until use.

The study was approved by the biomedical ethics committee of West China hospital (2019 [507]), and informed consent was obtained from each patient.

### 2.2. Dedifferentiation of PTC Cell Lines by ^131^I Pretreatment

Dedifferentiation transformation of PTC cell lines was performed as previously described [17]. RAIU and Western blot analyses of NIS (1:1500; Chemicon International, Hofheim, Germany), TPO (1:500; Thermo Fisher Scientific Inc., Fremont, CA, USA), and TSHR (1:1000; Santa Cruz Biotechnology, Santa Cruz, CA, USA) were also performed to characterize the dedifferentiation of the cell lines. The RAIU assay was evaluated by a gamma counter. The cells were incubated at 37 °C for 30 min with 1 mL Hank’s Balanced Salt Solution (HBSS) containing 0.1 μCi Na^125^I and 1 μmol/L NaI. At the end of incubation, we washed the cells with cold HBSS for 1 min (×3) and transferred them into vials for counting.

### 2.3. Isolation and Identification of Exosomes

After 48 h of culture with FBS-depleted medium (to avoid interference from FBS-derived exogenous exosomes), exosomes were isolated from BCPAP R^+^/BCPAP and K1 R^+^/K1 cell supernatants by ultracentrifugation. The exosomes were characterized by Western blot analysis of the exosome-specific markers Alix (sc-53540, Santa Cruz, CA, USA), CD9 (60232-I-Ig, Proteintech, Rosemont, IL, USA), TSG101 (sc-136111, Santa Cruz, CA, USA), and calnexin (10427-2-AP, Proteintech, Rosemont, IL, USA). The exosomes were also identified by nanoparticle tracking analysis (NTA) (ZetaView 8.02.28) and transmission electron microscopy (TEM) (H-7650).

### 2.4. Total RNA Extraction and RNA Analyses

Total RNA was extracted from exosomes using TRIzol reagent (Invitrogen Life Technologies, Carlsbad, CA, USA) according to the manufacturer’s instructions. RNA purity was tested using a Nano-Photometer spectrophotometer (IMPLEN, Westlake Village, CA, USA). The eligible samples were used for high-throughput RNA-Seq of miRNAs. The miRNA expression levels were estimated by transcript per million (TPM), and differentially expressed miRNAs were filtered by |log2 (fold change)| > 1 and *p* < 0.05. The NIS-related miRNAs were then identified by the TargetScan and miRWalk databases. A Venn diagram shows shared differentially expressed miRNAs between NGS and NIS-related miRNAs which was selected for the later experiments.

### 2.5. qRT-PCR of miRNAs

The extraction of total RNA from serum exosomes was performed with an ExoReasy Serum/Serum Maxi Kit (QIAGEN, Dusseldorf, Germany). The MiRCURY LNA^TM^ miRNA PCR Starter Kit was used for qRT-PCR (QIAGEN, Dusseldorf, Germany), and miR-103a-3p was used as an internal control. The expression level was calculated by the 2^−ΔΔCT^ method. The primers were as follows: miR-15a-5p (Cat. #YP00204066), miR-500a-5p (Cat. #YP00204794), miR-1296-5p (Cat. #YP00206020), miR-3120-5p (Cat. #YP02108916), miR-3911 (Cat. #YP02104224), miR-6842-3p (Cat. #YP02115310), and miR-6858-5p (Cat. #YP02112076).

### 2.6. Fluorescence In Situ Hybridization (FISH)

To investigate the differences of miR-1296-5p level in tumor cells between PTC patients with non-^131^I-avid metastases and those with ^131^I-avid metastases, FISH was performed on paraffin-embedded sections by using the 5′- and 3′- digoxigenin-labeled locked nucleic acid probe complementary to the mature miR-1296-5p according to the manufacturer’s instructions. The miRNA signal was detected with the Alexa Fluor 488 Tyramide SuperBoost Kit (Invitrogen, Thermo Fisher Scientific, CA, USA) following the manufacturer’s directions.

### 2.7. The miRNA Transfections

PTC cells were seeded into six-well plates and cultured for 24–48 h until 50–60% confluent, then transiently transfected with corresponding miRNA control and miRNA mimics using a Lipofectamine 3000 Transfection Kit (Invitrogen) according to the manufacturer’s instructions. After 48 h of miRNA transfection, the cells were harvested and used for further assays.

### 2.8. Luciferase Reporter Analysis

The sequences for NIS-mut and NIS-wt containing the putative binding sites of miRNA were augmented and then cloned into the pmIRGLO-control luciferase reporter vectors (Promega, Madison, WI, USA). The wt/mut vectors were co-transfected with miRNA control and miRNA mimics into 293T cells with Lipofectamine 2000 (Invitrogen). At 36 h after cell culture, cells were lysed, and luciferase activities were tested with Dual-Luciferase Reporter Assay System.

### 2.9. Statistical Analysis

We used the Statistical Package for the Social Sciences, version 20.0 (SPSS, Chicago, IL, USA) and GraphPad Prism version 8.0 (GraphPad Software, Inc., San Diego, CA, USA) for data analysis. The continuous data were presented as the mean ± standard deviation/standard error or median and compared using Student’s t-test or one-way ANOVA. The categorical variables were presented as the number and performed using the Chi-square test. A receiver operating characteristic (ROC) curve was constructed to determine the predictive efficacy, and a logistic regression model was used to determine the most relevant circulating exosomal miRNAs for RAIR PTC. The results were considered significant when the *p*-values were <0.05.

## 3. Results

### 3.1. Establishment of RAIR PTC Cell Lines

**Figure 1:**
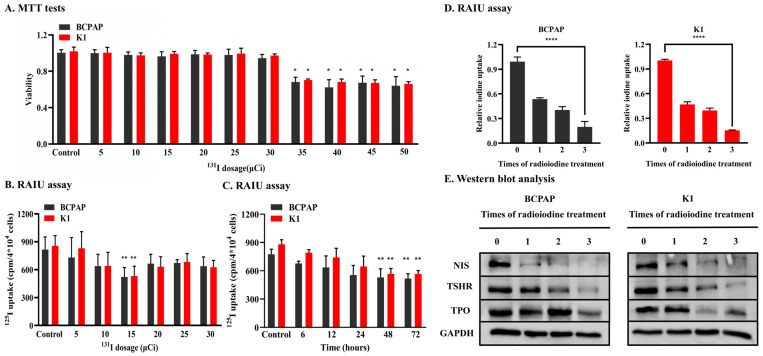
Establishment of radio-iodine refractory PTC cell lines. (**A**). MTT tests suggested BCPAP and K1 treated with ^131^I doses of more than 30 μCi showed toxicity in two cell lines. (**B**). RAIU of two cell lines incubated with differing doses of ^131^I for 12 h. 125I uptake in two cells lines with 15 μCi ^131^I decreased most significantly compared with that of the control. (**C**). RAIU of two cell lines incubated with 15 μCi ^131^I for different time periods. Compared with the control, ^125^I uptake of every experimental group gradually decreased. (**D**). RAIU were significantly decreased in treated BCPAP and K1 compared with that of the control. (**E**). The expression of the thyroid-specific protein (NIS, TPO, TSHR) were significant decreased in treated BCPAP and K1 compared with that of the control. * *p* < 0.05, ** *p* < 0.01, **** *p* < 0.0001.

In the MTT tests, the death rates of BCPAP and K1 cells treated with ^131^I at doses of 5 μCi to 30 μCi were similar to that of control cells (their absorbance was similar), but the toxicity was clearly observed in both cells at doses of more than 30 μCi (Figure 1A). BCPAP and K1 cells were then incubated with 5 μCi to 30 μCi ^131^I for 12 h to determine the optimal dose for subsequent experiments. The results showed that RAIU was significantly decreased at 15 μCi in both cell lines (Figure 1B). Thus, BCPAP and K1 cells were incubated with 15 μCi ^131^I for 6, 12, 24, 48 and 72 h. The RAIU assay showed that RAIU decreased gradually from 6 h after the start of incubation with ^131^I. After 48 h, the RAIU did not decrease (Figure 1C). Finally, BCPAP and K1 cells were incubated with 15 μCi ^131^I for 48 h 3 times (at intervals of one week) to establish RAIR PTC cell lines in our study. RAIU and Western blot assays revealed that ^125^I uptake (Figure 1D) and the expression of thyroid-specific proteins (NIS, TPO, TSHR) (Figure 1E) were significantly decreased in treated BCPAP and K1 cells. These results indicated that the RAIR PTC cell lines had been successfully established.

### 3.2. Identification and Verification of Candidate Exosomal miRNAs Associated with RAIR PTC

**Figure 2:**
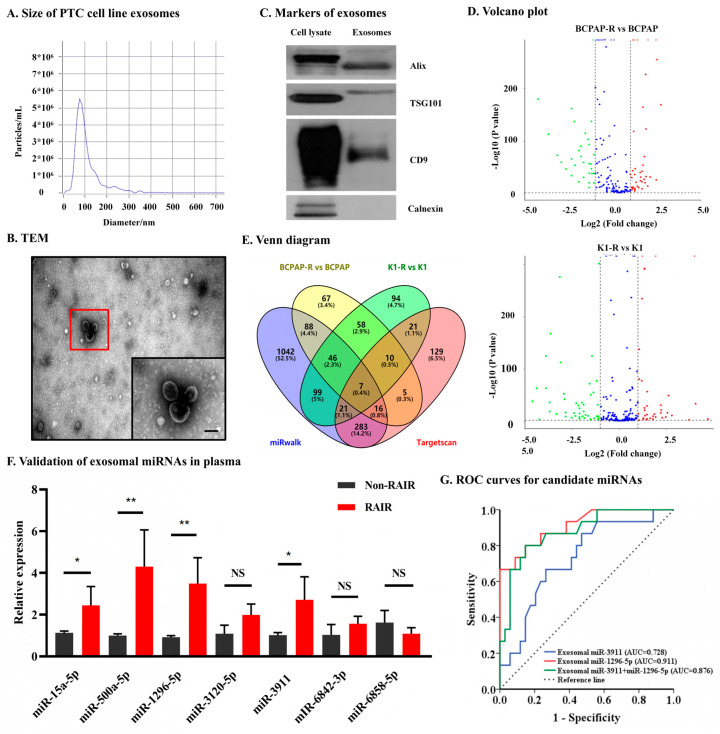
Identification and verification of candidate exosomal miRNAs associated with RAIR PTC. (**A**). NTA results suggested that exosomes were about 50–150 nm in diameter. (**B**). TEM images showed that exosomes were oval or bowl-shaped capsules without the nucleus. The red flame is the representative image. scale bare, 100 nm (**C**). exosomes markers Alix, CD9 and TSG101 were all detected and Calnexin, a negative marker of exosomes was absent in our isolated exosomes. (**D**). Differential expressed exosomal miRNA in treated PTC and parental cell lines. (**E**). A Venn diagram showed differentially expressed exosomal miRNAs (RAIR cell lines vs. parental cell lines) and miRNAs related to the NIS (predicted by miRwalk and Targetscan). (**F**). Validation of the differentially expressed selected plasma exosomal miRNAs in PTC patients with non-^131^I-avid metastases and ^131^I-avid metastases. (**G**). Receiver operating characteristic (ROC) curve of exosomal miR-1296-5p, miR-3911 and their combinations as a predictive marker for radio-iodine refractory PTC. NS, no significance, * *p* < 0.05, ** *p* < 0.01.

To identify differentially expressed miRNAs in exosomes between RAIR cell lines and parental cell lines, the exosome was isolated. The NTA analysis showed that the peak particle size was 80.3 nm (Figure 2A). The morphology of exosomes was evaluated by TEM, which showed that exosomes in the isolated fractions were oval or bowl-shaped (Figure 2B). Enrichment of the exosome markers Alix, CD9, and TSG101 was detected in the exosome-enriched fractions isolated from the cell supernatant. In contrast, calnexin, a negative marker of exosomes, was absent in our isolated exosome-enriched fraction samples (Figure 2C). Next, exosomal miRNA NGS was performed in the BCPAP R^+^/BCPAP group and K1 R^+^/K1 group (Figure 2D). In addition, the miRNAs related to the NIS (predicted by miRwalk and Targetscan) were selected due to the important role of NIS protein in iodine uptake. Ultimately, intersection analysis of the differentially expressed exosomal miRNAs (RAIR cell lines vs. parental cell lines) and miRNAs associated with NIS, five upregulated miRNAs and two downregulated miRNAs were identified (Figure 2E and Table 1).

Next, to verify above differentially expressed exosomal miRNAs in plasma specimen, 48 PTC patients with non-^131^I-avid metastases and 21 with ^131^I-avid metastases were enrolled at our institution. The demographic and clinical features of these patients are summarized in Table 2. The results of qRT-PCR confirmed the upregulation of exosomal miR-1296-5p and miR-3911 (all *p* < 0.05). However, the expression of the other five miRNAs either was not significantly different between the two groups of patents (miR-3120-5p, miR-6842-3p, miR-6858-5p) or showed the opposite results to the NGS results (miR-15a-5p, miR-500a-5p) (Figure 2F). Taken together, these results suggested that circulating exosomal miR-1296-5p and miR-3911 is significantly upregulated in patients with RAIR PTC.

### 3.3. Circulating Exosomal miR-1296-5p Is a Good Biomarker and an Independent Risk Factor for RAIR PTC

The diagnostic power of circulating exosomal miR-1296-5p, miR-3911 and their combination in RAIR PTC were next determined by ROC curve. As shown in Figure 2G, circulating exosomal miR-1296-5p and miR-3911 achieved areas under the curve (AUC) of 0.911 and 0.728, respectively. The best cutoff value of 2^−ΔΔCT^ for circulating exosomal miR-1296-5p and miR-3911 in predicting RAIR PTC was 1.9 and 1.2, respectively. The circulating exosomal miR-1296-5p had the highest sensitivity and specificity (72.2% and 93.6%, respectively) in comparison with miR-3911 (sensitivity and specificity of 66.7% and 71.4%, respectively). Additionally, combining circulating exosomal miR-1296-5p and miR-3911 can significantly improve the diagnostic power of miR-3911 by increasing the AUC to 0.876 with a sensitivity and specificity of 80.0% and 85.3%, respectively, but did not improve the diagnostic power of miR-1296-5p. To further identify the most relevant circulating exosomal miRNAs to RAIR PTC, logistic regression was performed, and the results revealed that circulating exosomal miR-1296-5p is an independent risk factor (Table 3). These results indicated that circulating exosomal miR-1296-5p may be involved in the pathogenesis of RAIR PTC.

### 3.4. miR-1296-5p Directly Targets the Downstream Gene of NIS

**Figure 3:**
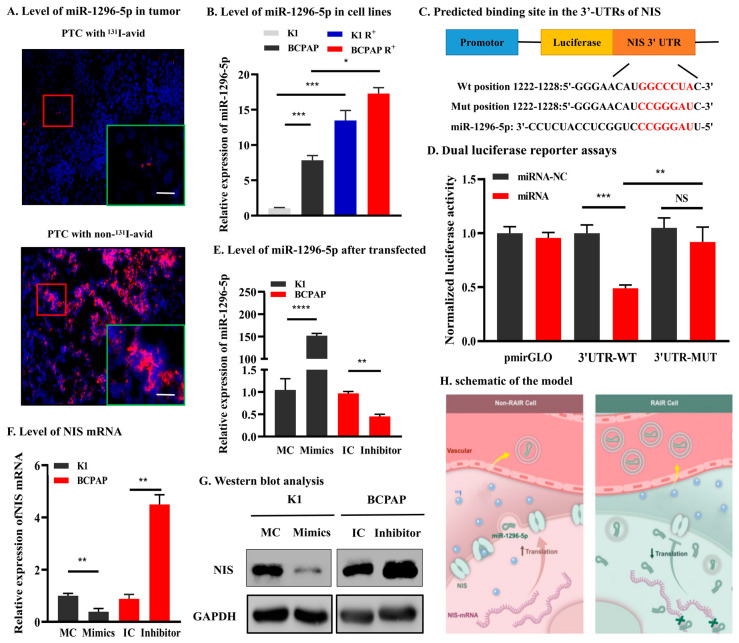
NIS is a direct target of miR-1296-5p. (**A**). The miR-1296-5p was abundantly detected in RAIR PTC tissue and cells. (**B**). Predicted binding site in the 3′-UTRs of NIS. The red flame is the representative image. scale bare, 20 μm (**C**). Dual luciferase reporter assays in 293T cells. (**D**). The basal expression levels of miR-1296-5p in K1 and BCPAP cells. (**E**). MiR-1296-5p expression was significantly upregulated after transfection with miR-mimic but downregulated after transfection with miR-inhibitor. (**F**,**G**). qRT-PCR and Western blotting showed that miR-1296-5p could inhibit NIS expression both in mRNA and protein levels. (**H**). schematic of the model. * *p* < 0.05, ** *p* < 0.01, *** *p* < 0.001, **** *p* < 0.0001.

Mechanism experiments were further conducted to ensure the association between miR-1296-5p and NIS in PTC. The results showed that miR-1296-5p was abundantly detected in RAIR PTC tissue and cells (Figure 3A,B). Furthermore, we identified a putative binding site of miR-1296-5p in the 3′-UTR of NIS (Figure 3C). Dual-luciferase reporter assay revealed that miR-1296-5p over-expression significantly decreased the luciferase reporter activity of the NIS wild-type vector in 293T cells (Figure 3D). Next, miR-1296-5p over-expression models in K1 cells and miR-1296-5p knockdown models in BCPAP cells were established. The qRT-PCR indicated that the level of miR-1296-5p in K1 cells was significantly up-regulated while the level of miR-1296-5p in BCPAP cells was significantly down-regulated after transinfected with the mimics or inhibitor (Figure 3E). Following this, qRT-PCR and Western blot showed that the NIS mRNA and protein levels were substantially decreased after ectopic over-expression of miR-1296-5p in K1 cells and reduced of miR-1296-5p remarkably promoted NIS expression in BCPAP cells compared with that in MC and IC groups, respectively (Figure 3F,G). Taken together, we confirmed that NIS is the target gene of miR-1296-5p.

## 4. Discussion

The most common cause of death associated with TC is metastatic disease, and the ultimate clinical goal of treatment is to improve survival. At present, radioiodine remains a mainstay of therapy for these patients. Unfortunately, 2% to 5% of DTC lose their differentiated phenotype, resulting in impaired iodine trapping, radioiodine resistance, and a poor prognosis [18].

The NIS is the sole human transporter responsible for iodide uptake, and its exploitation represents the first and most specifically targeted internal radiation therapy available. It has been shown that the ability of NIS to transport radioisotopes mainly depends on its expression level and localization in the cell. Unfortunately, BRAF mutation, occurring in approximately 60–70% PTC, can significantly influence NIS activity, including reducing its expression levels and/or inducing abnormal localization (decreased or absent from the plasma membrane), which is considered as the principal mechanisms behind RAIR PTC [19]. Hence, numerous studies have attempted to enhance the expression and function of NIS in RAIR TC patients, thereby re-sensitizing tumors to radioiodine therapy. The previous studies showed that ^131^I uptake of TC was improved by treated with retinoids [20], peroxisome proliferator activator receptor-γ agonists [21], BRAF and ERK1/2 inhibitors [22], and histone deacetylase inhibitors [23]. Multiple biologically targeted drugs, such as Lenvatinib [24] and dabrafenib [13,25], have been evaluated in phase I, II, and III trials and showed promising responses and/or disease stabilization. However, issues of toxicity and drug resistance remain.

Exosomes, a kind of small extracellular vesicles, are derived from the endosomal compartment and range in size from 30 to 150 nm in diameter, which contain a selection of mRNA, protein, DNA, and miRNA “cargo” molecules [26]. MiRNAs, an endogenous 21–23 nt small noncoding RNAs capable of controlling gene expression through posttranscriptional regulation, are essential for embryo development [27], oncogenesis [28], immune regulation [29], and other biological processes [30]. During disease, aberrantly expressed miRNAs, which are associated with higher disease activity that circulated miRNAs in diseased cells that are released into the circulation and the lipid bilayer membrane structure of exosomes protects miRNAs from degradation by enzymes in the circulation system [31]. Few studies have addressed the direct role of exosomal miRNAs in RAIR PTC.

Our study for the first time presented exosome-derived miRNA profiling of dedifferentiated PTC cell lines by ^131^I pretreatment and untreated PTC cell lines. The data revealed that exosomal miR-1296-5p were significantly upregulated in dedifferentiated PTC cell lines, which was validated by qRT-PCR in circulating exosomes between PTC patients with non-^131^I-avid metastases and those with ^131^I-avid metastases. Additionally, the data displayed that miR-1296-5p upregulation could affect the relative gene expression of NIS. Based on our findings, we speculated that PTC cells will generate more miR-1296-5p during ^131^I pretreatment so that they can secrete more miR-1296-5p into the circulatory system via exosomes and reduce NIS protein levels by targeting NIS mRNA (Figure 3H).

MiRNA-1296-5p, a novel cancer-related miRNA, plays important roles in regulating cancer cells across several cancer types, such as hepatocellular carcinoma, gastric cancer, and breast cancer. It has been reported that miR-1296-5p can act as a tumor suppressor in miRNA in reproductive system tumors and has ability of decreasing the proportion of S phase cells in prostate cancers [32]. In cervical cancer, Liu et al. uncovered that miR-1296-5p can regulate PIM1-STAT3 signaling pathway to induce apoptosis of tumor [33]. In Zang’s study, Circ-0000517 accelerated hepatocellular carcinoma progression through upregulation of TXNDC5 by sponging miR-1296-5p [34]. Yan’s study and Xia’s study demonstrated that miR-1296-5p inhibits the migration and invasion of gastric cancer cells [35,36]. A similar tumor-suppressive function was also found in osteosarcoma [37]. To the best of our knowledge, the role of exosomal miR-1296-5p in RAIR PTC was first reported in our study.

Recently, human primary cell cultures have been established as monolayer cultures and investigated for their biological behavior. Moreover, in the past, primary TC cells could be collected only through surgical biopsies, while recently human primary cell cultures can be established also from samples of fine-needle aspiration cytology from aggressive TC [38]. Compared with cell lines, primary cells can keep the characteristic features of the primary tumor, thus furthering progress regarding use of primary differentiated and dedifferentiated TC cells could be made in this direction to find exosomal miRNAs that may contribute to the development of RAIR.

Inevitably, some limitations of the current study should be mentioned. First, although the relative expression of exosomal miR-3120-5p, miR-6842-3p, and miR-6858-5p were not significantly different between PTC patients with iodine avid and non-avid metastases, the small sample size in the present study may have biased the results. In this regard, studies of larger cohorts are still required to support our findings. Second, the function (especially iodine uptake ability) and mechanisms underlying the altered expression levels of exosomal miR-1296-5p still need to be further investigated.

In conclusion, our findings suggest that circulating exosomal miRNAs, particularly miR-1296-5p, may be involved in the regulation of RAIR PTC by directly targeting NIS, which may provide new strategies for the diagnosis and treatment in RAIR PTC.

## Figures and Tables

**Table 1:** Seven candidate miRNAs which were related to NIS.

Exosomal miRNAs	Log2. FC (B)	*p*-Value	Log2. FC (K)	*p*-Value	Description
miR-15a-5p	−6.5031	<0.001	−4.2368	0.002	Down
miR-500a-5p	−2.9256	<0.001	−5.8217	<0.001	Down
miR-1296-5p	6.1642	<0.001	3.0664	0.007	Up
miR-3120-5p	5.1564	<0.001	3.7183	0.002	Up
miR-3911	6.7649	<0.001	3.5621	0.002	Up
miR-6842-3p	4.2663	<0.001	6.5256	<0.001	Up
miR-6858-5p	5.2635	<0.001	2.7625	0.002	Up

NIS: Na^+^/I^−^ symporter; FC: fold change; B: BCPAP R^+^ vs. BCPAP; K: K1 R^+^ vs. K1.

**Table 2:** Clinical characteristics of PTC patients with non-131I-avid and 131I-avid metastases.

Clinical Characteristics	^131^I-avid (*n* = 48)	Non-^131^I-avid (*n* = 21)	*p*-Value
Age (mean ± SD, years)	45.2 ± 12.1	47.7 ± 18.3	0.514
≥55/<55	9 (18.8%)/39 (81.2%)	6 (28.6%)/15 (71.4%)	0.363
Gender (male/female)	16 (33.3%)/32 (66.7%)	9 (42.9%)/12(57.1%)	0.449
BMI (mean ± SD, kg/m2)	23.0 ± 3.0	23.3 ± 4.5	0.722
Nodular goiter (yes/no)	27 (56.3%)/21 (43.7%)	8 (38.1%)/13 (61.9%)	0.165
Hashimoto’s thyroiditis (yes/no)	8 (16.7%)/40 (83.3%)	4 (19.0%)/17 (81.0%)	0.811
Graves’s disease (yes/no)	4 (8.3%)/44 (91.7%)	0 (0.0%)/21 (100.0%)	0.306
Multifocality (yes/no)	16 (33.3%)/32 (66.7%)	8 (38.1%)/13 (61.7%)	0.702
T stage (T1-T2/T3-T4)	4 (8.3%)/44 (91.7%)	1 (4.8%)/20 (95.2%)	0.553
*n* stage (N0/N1)	7 (14.6%)/41 (85.4%)	1 (4.8%)/20 (95.2%)	0.420
RAI dose (median, uci)	100 (100–150)	100 (100–200)	0.698

PTC: papillary thyroid cancer; SD: standard deviation; BMI: body mass index; RAI: radioiodine.

**Table 3:** Univariate and multiple analysis for difference miRNAs.

miRNAs	Univariate		Multivariate	
	OR (95%CI)	*p* Value	OR (95%CI)	*p* Value
miR-1296-5p	10.233 (3.164–33.091)	<0.001 *	12.319 (2.853–53.192)	0.001 *
miR-3911	1.765 (0.796–3.916)	0.162	1.092 (0.523–2.277)	0.815

CI: confidence interval; OR: Odds ratio; * statistically significant difference.

## Data Availability

The raw data regarding RNA sequencing in the study could be found in online repositories: https://www.ebi.ac.uk/arrayexpress/experiments/E-MTAB-10121 (accessed on 17 February 2021).

## Abbreviations

AUC = areas under the curve; FISH = fluorescence in situ hybridization; miRNAs = microRNAs; NGS = next-generation sequencing; NIS = Na^+^/I^−^ symporter; NTA = nanoparticle tracking analysis; PTC = papillary thyroid carcinoma; qRT-PCR = quantitative real-time PCR; RAIR = radioiodine refractory; RAIU = radioiodine uptake; ROC = receiver operating characteristic; TC = Thyroid cancer; TEM = transmission electron microscopy; TKIs = tyrosine kinase inhibitors; TSH = thyroid stimulating hormone.
